# Nurse-directed Preventative Management of Atrial Fibrillation: Is it Feasible?

**DOI:** 10.19102/icrm.2019.100904

**Published:** 2019-09-15

**Authors:** Mary E. Huntsinger, Rahul N. Doshi

**Affiliations:** ^1^Division of Cardiology, Keck School of Medicine, University of Southern California, Los Angeles, CA, USA

**Keywords:** Allied professionals, atrial fibrillation, lifestyle modification, nursing

Atrial fibrillation (AF) is the most common arrhythmia seen in clinical practice. The symptoms often associated with AF, such as dyspnea, fatigue, and palpitations, may be associated with other cardiac conditions such as heart failure, which may make recognition of the condition in certain patients more difficult. This can, in turn, cause a delay in the performance of a timely evaluation and treatment initiation. There are multiple identifiable risk factors for AF including hypertension, obesity, smoking, diabetes, sleep apnea, and alcohol intake. An enduring question is how and when is it best to address these risk factors. To date, there is a scarcity of evidence-based studies demonstrating preventive interventions that reduce or prevent the occurrence of AF.

Today, rhythm control strategies for AF are limited in efficacy. Antiarrhythmic drug (AAD) therapy pros and cons have been well-established. The efficacy of AADs is at best 52%, based on a meta-analysis of 32 studies.^1^ Electrical cardioversion has a documented 40% to 60% recurrent AF rate within three months and up to a 60% to 80% recurrence rate within one year.^2^ Catheter ablation has a single-procedure success rate of 50% to 64%, which increases to 65% to 77% with repeat ablations and with the patient off AADs.^1^ Given the limitations of current therapy, interventions that increase the efficacy of treatment are of obvious importance.

Several studies have shown that aggressive risk factor reduction improves the long-term outcomes of catheter ablation. The Aggressive Risk Factor Reduction Study for AF and Implications for the Outcome of Ablation (ARREST-AF) cohort study found that a structured physician-directed risk factor and weight management program results in the significant improvement of long-term outcomes, including specifically significant improvement in left atrial volumes and left ventricular hypertrophy, leading to a lower risk of AF. Risk factor management included the control of blood pressure, weight, lipid levels, sleep-disordered breathing, and glycemic levels in addition to catheter ablation.^3^ The Long-term Effect of Goal-directed Weight Management in an AF Cohort (LEGACY) study separately showed that AF burden, reductions in symptom severity, and long-term freedom from AF were dose-dependent on the amount of weight lost.^4^ Another trial, the Cost-effectiveness and Clinical Effectiveness of Risk Factor Management Clinic in AF (CENT Study), revealed that a structured physician-directed aggressive risk factor modification program was clinically effective and cost-effective in the management of AF. The risk factor management strategy was associated with higher upfront costs due to increased clinic visits, but, in the long term, it reduced overall costs by decreasing the number of ablations needed.^5^ These trials indicate that risk factor reduction or control lowers AF and costs. However, most relevant studies to date have been physician-directed and resource-intensive. Given a relative physician shortage, alternate ways of delivering care should be explored. One of the few nonphysician studies from the University of Pennsylvania developed a program addressing obesity and obstructive sleep apnea (OSA) in AF patients in their electrophysiology outpatient clinics. This nurse-led program achieved greater success rates in the included patients with regard to weight reduction and OSA diagnosis and treatment.^6^ It has been previously demonstrated that involving nurses in heart failure programs as well as hospital education programs decreases patient readmission rates and costs and improves the quality of life of patients.^7^

The pilot trial by Hickey et al.^8^ is timely in that it addresses a multitude of issues related to patient education and compliance with recommended treatments of AF. Nurses are effective at educating patients and following up with those with chronic conditions, as evidenced in heart failure and inflammatory bowel disease populations. Nurses have been documented to promote decreased visits through education and alternate forms of communication.^7,9^ For certain medical conditions, patients felt that telemedicine was a convenient alternative to traditional in-office care without compromising the quality of care.^9^

This study had participants utilizing the Life’s Simple 7^®^ assessment for the identification of goals for lifestyle and self-care management behaviors and the My AFib Symptom Tracker to monitor their symptom details, both from the American Heart Association (Dallas, TX, USA). Participants selected lifestyle modification areas from the Life’s Simple 7^®^ interface that they believed they could act upon over the study period. For six months, the participants participated in biweekly video chats with a cardiac nurse via the portal, representing the behavioral intervention of motivational interviewing. The cardiac nurse reviewed and discussed lifestyle modifications and self-management strategies incorporated by the participant since the previous session. The sessions were guided by individual cardiac goals and the patient’s progress. Participants documented events of note on the My AFib Experience Symptom Tracker and identified whether symptoms occurred at rest or during exercise as well as the severity and frequency of the occurrences. This helped to guide patients to better recognize the association of symptoms and triggers with AF rather than other cardiac conditions.

The results of the 53 study participants demonstrated that 98% liked having a dedicated nurse working with them on a biweekly basis to improve their cardiac health and AF recognition. Some participants reported a desire to have more frequent contact with the nurse, while 86% of participants chose increasing physical activity as the primary goal they could improve upon and 65% decided to include improving their diet in their goals. On average, there was a 3-lb decrease in weight and a 5-mmHg decrease in systolic blood pressure from baseline. The overall reported compliance rate was 65% for the entire cohort. Participants who received the intervention achieved a mean one-point improvement in their Life’s Simple 7^®^ score over the intervention period, while 70% of participants improved their Life’s Simple 7^®^ score by two or more points from baseline to six months. Further, 81% of participants reported that the intervention enhanced accessibility, eliminated extra in-person visits, provided health information for achieving “my health goals,” and felt the digital sessions to be as good as in-person meetings with the nurse practitioner (NP). They reported the “human connection” provided by the nurse combined with the motivational interviewing to be the most essential elements in helping them stay on course with sustaining a healthy lifestyle and with overall AF management.

This trial successfully demonstrated the efficacy of NPs regarding communicating and following patients through a digital portal and helping them to stay motivated and focused. In addition, providing education to and tracking study participants, especially those with loop recorders, led patients to better be able to identify and distinguish their AF symptoms and associated triggers. The authors also found that regular contact between patients and the cardiology NP helped with medication compliance and using proper medication strategies, especially in terms of antiarrhythmics and anticoagulation agents.

One limitation of the study is its small sample size of 53 individuals. It was also a single-arm study without a control arm and was conducted at a single center, all of which were identified by the authors as limitations. The results could be attributed to a placebo effect or treatment bias, including the mere attention given to participants.^10^ Another limitation to this study is that it did not address some major modifiable risk factors such as sleep apnea and alcohol consumption, both of which have been documented to contribute to and impact AF burden. Also, this study was only six months long and so the longer-term outcomes remain to be seen.

That being said, this study is timely as health care providers continue to look for alternate ways of providing care and due to its preventive focus. Given the limitations of our current treatment strategies for AF, lifestyle modification to increase overall treatment success is both plausible and feasible. Also, given the ongoing physician shortage and unique skillsets that nurses can provide, the focus on nurse-driven lifestyle modification interventions is not just optimistic but necessary. We eagerly look forward to the next phase of this study.
